# (*E*)-4-[4-(Diethyl­amino)­benzyl­idene­ammonio]­benzene­sulfonate

**DOI:** 10.1107/S1600536812026402

**Published:** 2012-06-20

**Authors:** Pumsak Ruanwas, Suchada Chantrapromma, Hoong-Kun Fun

**Affiliations:** a Department of Chemistry and Center of Excellence for Innovation in Chemistry, Faculty of Science, Prince of Songkla University, Hat-Yai, Songkhla 90112, Thailand; b Crystal Materials Research Unit, Department of Chemistry, Faculty of Science, Prince of Songkla University, Hat-Yai, Songkhla 90112, Thailand; c X-ray Crystallography Unit, School of Physics, Universiti Sains Malaysia, 11800 USM, Penang, Malaysia

## Abstract

The title compound, C_17_H_20_N_2_O_3_S, synthesised from sulfanilic acid and 4-diethyl­amino­benzaldehyde, crystallized out as a zwitterion with the central N atom protonated. The zwitterion exists in an *E* conformation with respect to the C=N double bond. The dihedral angle between the benzene rings is 37.57 (5)°. In the crystal, the zwitterions are linked into a tape along the *a* axis by N—H⋯O hydrogen bonds. The crystal structure is further stabilized by weak C—H⋯O inter­actions and π–π inter­actions with a centroid–centroid distance of 3.8541 (6) Å. An O⋯O [2.8498 (11) Å] short contact is present.

## Related literature

For bond-length data, see: Allen *et al.* (1987 ▶). For related structures, see: Banu & Golzar Hossain (2006 ▶); Yeap *et al.* (2010 ▶). For background and applications to sulfanilic acids, see: Chanawanno *et al.* (2010 ▶); Hussain *et al.* (2009 ▶); Kim *et al.* (2011 ▶); King (1991 ▶); Taylor *et al.* (2006 ▶). For the stability of the temperature controller used in the data collection, see: Cosier & Glazer (1986 ▶).
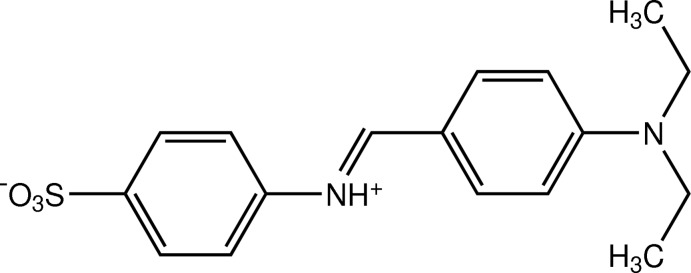


## Experimental

### 

#### Crystal data

C_17_H_20_N_2_O_3_S
*M* *_r_* = 332.42
Triclinic, 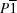

*a* = 8.1227 (1) Å
*b* = 8.8745 (1) Å
*c* = 12.4070 (2) Å
α = 69.386 (1)°
β = 72.442 (1)°
γ = 75.509 (1)°
*V* = 787.63 (2) Å^3^
*Z* = 2
Mo *K*α radiation
μ = 0.22 mm^−1^
*T* = 100 K
0.45 × 0.22 × 0.14 mm

#### Data collection

Bruker APEXII CCD area-detector diffractometer
Absorption correction: multi-scan (*SADABS*; Bruker, 2005 ▶) *T*_min_ = 0.906, *T*_max_ = 0.969
24759 measured reflections
5685 independent reflections
5040 reflections with *I* > 2σ(*I*)
*R*_int_ = 0.024

#### Refinement

*R*[*F* ^2^ > 2σ(*F* ^2^)] = 0.034
*wR*(*F* ^2^) = 0.097
*S* = 1.04
5685 reflections
214 parameters
H atoms treated by a mixture of independent and constrained refinement
Δρ_max_ = 0.77 e Å^−3^
Δρ_min_ = −0.40 e Å^−3^

### 

Data collection: *APEX2* (Bruker, 2005 ▶); cell refinement: *SAINT* (Bruker, 2005 ▶); data reduction: *SAINT*; program(s) used to solve structure: *SHELXTL* (Sheldrick, 2008 ▶); program(s) used to refine structure: *SHELXTL*; molecular graphics: *SHELXTL*; software used to prepare material for publication: *SHELXTL* and *PLATON* (Spek, 2009 ▶).

## Supplementary Material

Crystal structure: contains datablock(s) global, I. DOI: 10.1107/S1600536812026402/is5152sup1.cif

Structure factors: contains datablock(s) I. DOI: 10.1107/S1600536812026402/is5152Isup2.hkl

Supplementary material file. DOI: 10.1107/S1600536812026402/is5152Isup3.cml

Additional supplementary materials:  crystallographic information; 3D view; checkCIF report

## Figures and Tables

**Table 1:** Hydrogen-bond geometry (Å, °)

*D*—H⋯*A*	*D*—H	H⋯*A*	*D*⋯*A*	*D*—H⋯*A*
N1—H1*N*1⋯O3^i^	0.865 (18)	2.466 (17)	3.0309 (12)	123.6 (13)
N1—H1*N*1⋯O3^ii^	0.865 (18)	2.076 (18)	2.8989 (12)	158.7 (16)
C7—H7*A*⋯O2^iii^	0.93	2.39	3.2719 (13)	157
C13—H13*A*⋯O3^ii^	0.93	2.40	3.3002 (13)	164
C14—H14*A*⋯O1^iv^	0.97	2.58	3.4805 (14)	155

Symmetry codes: (i) 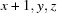; (ii) 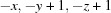; (iii) 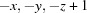; (iv) 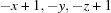.

**Fig. 1.**
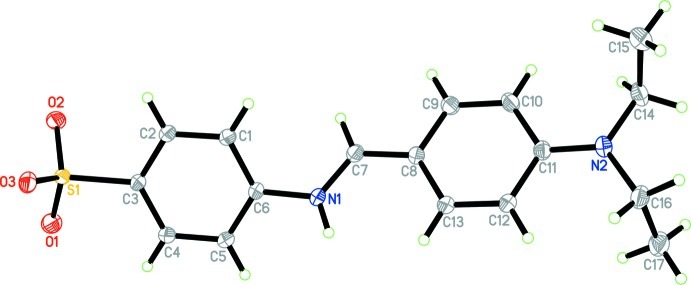
The molecular structure of the title compound, showing 60% probability displacement ellipsoids and the atom-numbering scheme.

**Fig. 2.**
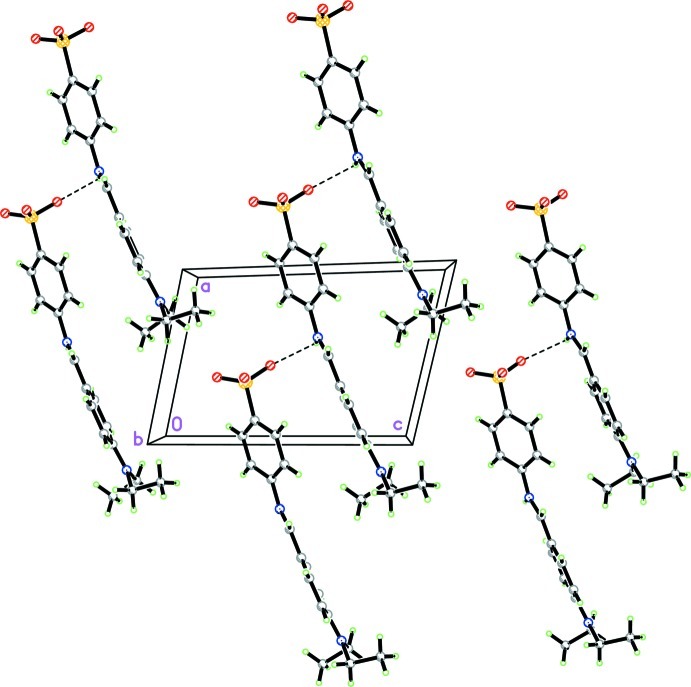
The crystal packing of the title compound viewed approximately along the b axis, showing the tape structures running along the a axis. Hydrogen bonds are shown as dashed lines.

## supplementary crystallographic information

### Comment

Benzenesulfonic acid is a very strong acid and coupled with aromatic components are interesting chemical reagents (King, 1991). Many of these combinations exhibit pharmaceutical and biological activities (Chanawanno *et al.*, 2010; Taylor *et al.*, 2006) and were also used as whitening reagents (Hussain *et al.*, 2009) and fluorescence sensors (Kim *et al.*, 2011). The title compound (I) was synthesized on account of its fluorescence property. It was found that (I) shows solid state fluorescence with the maximum emission at 625 nm when was excited at 400 nm. Herein the synthesis and crystal structure of (I) are reported.

In Fig. 1, the molecule of (I), C_17_H_20_N_2_O_3_S, crystallized out as a zwitterion with the N1 atom protonated. The hydrogen is more preferably attached to the nitrogen atom due to the stronger basicity of NH_2_ group compared to the SO_3_^-^ substituent (Banu & Golzar Hossain, 2006; Yeap *et al*., 2010). The zwitterion exists in an *E* configuration with respect to the central C═N double bond [1.3146 (12)°] with the torsion angle C6–N1–C7–C8 being 172.99 (9)°. The molecule is twisted with the dihedral angle between the two benzene rings being 37.57 (5) °. The two ethyl groups of diethylamino are out of its bound benzene ring plane with the torsion angles of C11–N2–C14–C15 = 84.88 (12)° and C11–N2–C16–C17 = 86.61 (12)° and they are oriented in oppositional directions to each other (Fig. 1). The bond distances agree with the literature values (Allen *et al.*, 1987) and are comparable with the related structure (Yeap *et al.*, 2010).

In the crystal packing (Fig. 2), the zwitterions are linked by intermolecular N—H···O hydrogen bonds (Table 1) into tapes along the *a* axis. The crystal is stabilized by intermolecular N—H···O hydrogen bonds and weak C—H···O interactions (Table 1). A π–π interaction with the distance of *Cg*_1_···*Cg*_1_^ii^ = 3.8541 (6) Å was observed; *Cg*_1_ is the centroid of C1–C6 benzene ring. An O···O^v^ short contact [2.8498 (11) Å; symmetry code (v) = -1 - *x*, 1 - *y*, 1 - *z*] was presented.

### Experimental

Sulfanilic acid (0.5 g, 2.8 mmol) was dissolved in water (10 ml) and then 4-diethylaminobenzaldehyde (0.5 g, 2.8 mmol) was added. The mixture was refluxed at 120 °C for 2 h. The precipitate was filtered, washed with water and purified by recrystallization from ethanol to afford the compound I (yield 79%). Yellow block-shaped single crystals of the title compound suitable for *X*-ray structure determination were formed from recrystallization from ethanol by the slow evaporation of the solvent at room temperature after a week (m.p. 540–541 K).

### Refinement

Amide H atom was located from a difference map and isotropically refined. The remaining H atoms were positioned geometrically and allowed to ride on their parent atoms, with d(C—H) = 0.93 Å for aromatic and CH, 0.97 Å for CH_2_ and 0.96 Å for CH_3_ atoms. The *U*_iso_ values were constrained to be 1.5*U*_eq_ of the carrier atom for methyl H atoms and 1.2*U*_eq_ for the remaining H atoms. A rotating group model was used for the methyl groups.

### Crystal data

**Table:**

C_17_H_20_N_2_O_3_S	*Z* = 2
*M**_r_* = 332.42	*F*(000) = 352
Triclinic, *P*1	*D*_x_ = 1.402 Mg m^−^^3^
Hall symbol: -P 1	Melting point = 540–541 K
*a* = 8.1227 (1) Å	Mo *K*α radiation, λ = 0.71073 Å
*b* = 8.8745 (1) Å	Cell parameters from 5685 reflections
*c* = 12.4070 (2) Å	θ = 1.8–32.5°
α = 69.386 (1)°	µ = 0.22 mm^−^^1^
β = 72.442 (1)°	*T* = 100 K
γ = 75.509 (1)°	Block, yellow
*V* = 787.63 (2) Å^3^	0.45 × 0.22 × 0.14 mm

### Data collection

**Table:**

Bruker APEXII CCD area-detector diffractometer	5685 independent reflections
Radiation source: sealed tube	5040 reflections with *I* > 2σ(*I*)
Graphite monochromator	*R*_int_ = 0.024
φ and ω scans	θ_max_ = 32.5°, θ_min_ = 1.8°
Absorption correction: multi-scan (*SADABS*; Bruker, 2005)	*h* = −12→12
*T*_min_ = 0.906, *T*_max_ = 0.969	*k* = −13→13
24759 measured reflections	*l* = −18→17

### Refinement

**Table:**

Refinement on *F*^2^	Primary atom site location: structure-invariant direct methods
Least-squares matrix: full	Secondary atom site location: difference Fourier map
*R*[*F*^2^ > 2σ(*F*^2^)] = 0.034	Hydrogen site location: inferred from neighbouring sites
*wR*(*F*^2^) = 0.097	H atoms treated by a mixture of independent and constrained refinement
*S* = 1.04	*w* = 1/[σ^2^(*F*_o_^2^) + (0.0477*P*)^2^ + 0.3239*P*] where *P* = (*F*_o_^2^ + 2*F*_c_^2^)/3
5685 reflections	(Δ/σ)_max_ = 0.001
214 parameters	Δρ_max_ = 0.77 e Å^−^^3^
0 restraints	Δρ_min_ = −0.40 e Å^−^^3^

### Special details

**Table:**

Experimental. The crystal was placed in the cold stream of an Oxford Cryosystems Cobra open-flow nitrogen cryostat (Cosier & Glazer, 1986) operating at 120.0 (1) K.
Geometry. All e.s.d.'s (except the e.s.d. in the dihedral angle between two l.s. planes) are estimated using the full covariance matrix. The cell e.s.d.'s are taken into account individually in the estimation of e.s.d.'s in distances, angles and torsion angles; correlations between e.s.d.'s in cell parameters are only used when they are defined by crystal symmetry. An approximate (isotropic) treatment of cell e.s.d.'s is used for estimating e.s.d.'s involving l.s. planes.
Refinement. Refinement of *F*^2^ against ALL reflections. The weighted *R*-factor *wR* and goodness of fit *S* are based on *F*^2^, conventional *R*-factors *R* are based on *F*, with *F* set to zero for negative *F*^2^. The threshold expression of *F*^2^ > σ(*F*^2^) is used only for calculating *R*-factors(gt) *etc*. and is not relevant to the choice of reflections for refinement. *R*-factors based on *F*^2^ are statistically about twice as large as those based on *F*, and *R*- factors based on ALL data will be even larger.

### Fractional atomic coordinates and isotropic or equivalent isotropic displacement parameters (Å^2^)

**Table:**

	*x*	*y*	*z*	*U*_iso_*/*U*_eq_	
S1	−0.35313 (3)	0.23999 (3)	0.67776 (2)	0.01330 (6)	
O1	−0.38256 (10)	0.25888 (10)	0.79395 (7)	0.02127 (16)	
O2	−0.37965 (10)	0.08274 (9)	0.67967 (7)	0.01962 (15)	
O3	−0.44670 (9)	0.37665 (9)	0.59896 (7)	0.01834 (14)	
N1	0.39588 (10)	0.29545 (10)	0.43756 (8)	0.01497 (15)	
N2	1.16999 (11)	0.27158 (11)	0.08368 (8)	0.01660 (16)	
C1	0.12637 (12)	0.20179 (11)	0.45697 (8)	0.01394 (16)	
H1A	0.1803	0.1600	0.3935	0.017*	
C2	−0.04759 (12)	0.18925 (11)	0.51483 (8)	0.01344 (16)	
H2A	−0.1106	0.1391	0.4898	0.016*	
C3	−0.12775 (11)	0.25131 (11)	0.60985 (8)	0.01259 (15)	
C4	−0.03462 (12)	0.32751 (12)	0.64768 (9)	0.01528 (17)	
H4A	−0.0885	0.3685	0.7115	0.018*	
C5	0.13909 (12)	0.34216 (12)	0.58977 (9)	0.01560 (17)	
H5A	0.2013	0.3944	0.6138	0.019*	
C6	0.21886 (11)	0.27794 (11)	0.49552 (8)	0.01322 (16)	
C7	0.50414 (12)	0.19565 (11)	0.38086 (8)	0.01395 (16)	
H7A	0.4650	0.1025	0.3867	0.017*	
C8	0.67488 (12)	0.21763 (11)	0.31182 (8)	0.01355 (16)	
C9	0.77809 (12)	0.09167 (12)	0.26404 (9)	0.01590 (17)	
H9A	0.7332	−0.0032	0.2817	0.019*	
C10	0.94259 (13)	0.10588 (12)	0.19236 (9)	0.01669 (17)	
H10A	1.0086	0.0198	0.1640	0.020*	
C11	1.01332 (12)	0.25146 (12)	0.16084 (8)	0.01427 (16)	
C12	0.91019 (12)	0.37647 (12)	0.21180 (9)	0.01533 (17)	
H12A	0.9551	0.4708	0.1956	0.018*	
C13	0.74652 (12)	0.36035 (12)	0.28410 (9)	0.01506 (17)	
H13A	0.6817	0.4442	0.3153	0.018*	
C14	1.28157 (13)	0.14184 (13)	0.03449 (10)	0.02023 (19)	
H14A	1.2715	0.0372	0.0948	0.024*	
H14B	1.4026	0.1575	0.0128	0.024*	
C15	1.23208 (15)	0.13989 (16)	−0.07380 (11)	0.0264 (2)	
H15A	1.3078	0.0536	−0.1030	0.040*	
H15B	1.2442	0.2425	−0.1343	0.040*	
H15C	1.1130	0.1223	−0.0524	0.040*	
C16	1.24190 (13)	0.42267 (13)	0.04692 (9)	0.01837 (18)	
H16A	1.1470	0.5145	0.0429	0.022*	
H16B	1.3185	0.4371	−0.0317	0.022*	
C17	1.34381 (15)	0.42088 (16)	0.13212 (10)	0.0247 (2)	
H17A	1.3825	0.5241	0.1080	0.037*	
H17B	1.4434	0.3356	0.1314	0.037*	
H17C	1.2698	0.4019	0.2107	0.037*	
H1N1	0.427 (2)	0.380 (2)	0.4405 (14)	0.030 (4)*	

### Atomic displacement parameters (Å^2^)

**Table:**

	*U*^11^	*U*^22^	*U*^33^	*U*^12^	*U*^13^	*U*^23^
S1	0.01089 (10)	0.01367 (10)	0.01646 (11)	−0.00400 (7)	−0.00018 (7)	−0.00702 (8)
O1	0.0193 (3)	0.0295 (4)	0.0174 (4)	−0.0095 (3)	0.0027 (3)	−0.0119 (3)
O2	0.0170 (3)	0.0154 (3)	0.0277 (4)	−0.0073 (2)	0.0013 (3)	−0.0102 (3)
O3	0.0130 (3)	0.0177 (3)	0.0254 (4)	−0.0014 (2)	−0.0054 (3)	−0.0078 (3)
N1	0.0111 (3)	0.0178 (4)	0.0183 (4)	−0.0050 (3)	−0.0009 (3)	−0.0085 (3)
N2	0.0127 (3)	0.0200 (4)	0.0166 (4)	−0.0038 (3)	0.0001 (3)	−0.0071 (3)
C1	0.0130 (4)	0.0163 (4)	0.0146 (4)	−0.0044 (3)	−0.0017 (3)	−0.0070 (3)
C2	0.0124 (3)	0.0145 (4)	0.0149 (4)	−0.0042 (3)	−0.0026 (3)	−0.0053 (3)
C3	0.0112 (3)	0.0126 (4)	0.0146 (4)	−0.0031 (3)	−0.0022 (3)	−0.0046 (3)
C4	0.0135 (4)	0.0174 (4)	0.0174 (4)	−0.0038 (3)	−0.0012 (3)	−0.0092 (3)
C5	0.0132 (4)	0.0181 (4)	0.0192 (4)	−0.0045 (3)	−0.0026 (3)	−0.0097 (3)
C6	0.0105 (3)	0.0143 (4)	0.0157 (4)	−0.0038 (3)	−0.0017 (3)	−0.0054 (3)
C7	0.0131 (4)	0.0155 (4)	0.0141 (4)	−0.0039 (3)	−0.0026 (3)	−0.0048 (3)
C8	0.0118 (3)	0.0156 (4)	0.0140 (4)	−0.0032 (3)	−0.0020 (3)	−0.0054 (3)
C9	0.0163 (4)	0.0147 (4)	0.0161 (4)	−0.0038 (3)	−0.0010 (3)	−0.0054 (3)
C10	0.0158 (4)	0.0155 (4)	0.0175 (4)	−0.0019 (3)	−0.0002 (3)	−0.0070 (3)
C11	0.0121 (4)	0.0169 (4)	0.0138 (4)	−0.0022 (3)	−0.0026 (3)	−0.0051 (3)
C12	0.0128 (4)	0.0160 (4)	0.0186 (4)	−0.0039 (3)	−0.0024 (3)	−0.0069 (3)
C13	0.0122 (4)	0.0162 (4)	0.0187 (4)	−0.0025 (3)	−0.0026 (3)	−0.0082 (3)
C14	0.0142 (4)	0.0241 (5)	0.0203 (5)	−0.0009 (3)	0.0008 (3)	−0.0098 (4)
C15	0.0235 (5)	0.0353 (6)	0.0236 (5)	−0.0071 (4)	0.0004 (4)	−0.0163 (5)
C16	0.0157 (4)	0.0231 (5)	0.0162 (4)	−0.0071 (3)	−0.0014 (3)	−0.0050 (4)
C17	0.0195 (4)	0.0374 (6)	0.0221 (5)	−0.0103 (4)	−0.0023 (4)	−0.0130 (4)

### Geometric parameters (Å, º)

**Table:**

S1—O1	1.4511 (8)	C8—C9	1.4148 (13)
S1—O2	1.4555 (7)	C8—C13	1.4152 (13)
S1—O3	1.4685 (8)	C9—C10	1.3714 (13)
S1—C3	1.7814 (9)	C9—H9A	0.9300
N1—C7	1.3146 (12)	C10—C11	1.4256 (13)
N1—C6	1.4206 (11)	C10—H10A	0.9300
N1—H1N1	0.867 (17)	C11—C12	1.4288 (13)
N2—C11	1.3514 (12)	C12—C13	1.3704 (13)
N2—C16	1.4654 (13)	C12—H12A	0.9300
N2—C14	1.4692 (13)	C13—H13A	0.9300
C1—C2	1.3910 (12)	C14—C15	1.5207 (16)
C1—C6	1.3958 (13)	C14—H14A	0.9700
C1—H1A	0.9300	C14—H14B	0.9700
C2—C3	1.3897 (13)	C15—H15A	0.9600
C2—H2A	0.9300	C15—H15B	0.9600
C3—C4	1.3942 (12)	C15—H15C	0.9600
C4—C5	1.3923 (13)	C16—C17	1.5207 (15)
C4—H4A	0.9300	C16—H16A	0.9700
C5—C6	1.3926 (13)	C16—H16B	0.9700
C5—H5A	0.9300	C17—H17A	0.9600
C7—C8	1.4128 (12)	C17—H17B	0.9600
C7—H7A	0.9300	C17—H17C	0.9600
			
O1—S1—O2	114.51 (5)	C8—C9—H9A	119.2
O1—S1—O3	112.95 (5)	C9—C10—C11	120.59 (9)
O2—S1—O3	112.08 (5)	C9—C10—H10A	119.7
O1—S1—C3	105.86 (4)	C11—C10—H10A	119.7
O2—S1—C3	106.04 (4)	N2—C11—C10	121.17 (9)
O3—S1—C3	104.40 (4)	N2—C11—C12	121.43 (9)
C7—N1—C6	123.77 (8)	C10—C11—C12	117.38 (8)
C7—N1—H1N1	121.8 (11)	C13—C12—C11	121.44 (8)
C6—N1—H1N1	114.4 (11)	C13—C12—H12A	119.3
C11—N2—C16	121.95 (8)	C11—C12—H12A	119.3
C11—N2—C14	122.25 (8)	C12—C13—C8	120.79 (9)
C16—N2—C14	115.76 (8)	C12—C13—H13A	119.6
C2—C1—C6	119.04 (9)	C8—C13—H13A	119.6
C2—C1—H1A	120.5	N2—C14—C15	112.15 (9)
C6—C1—H1A	120.5	N2—C14—H14A	109.2
C3—C2—C1	120.39 (8)	C15—C14—H14A	109.2
C3—C2—H2A	119.8	N2—C14—H14B	109.2
C1—C2—H2A	119.8	C15—C14—H14B	109.2
C2—C3—C4	120.24 (8)	H14A—C14—H14B	107.9
C2—C3—S1	118.97 (7)	C14—C15—H15A	109.5
C4—C3—S1	120.73 (7)	C14—C15—H15B	109.5
C5—C4—C3	119.90 (9)	H15A—C15—H15B	109.5
C5—C4—H4A	120.0	C14—C15—H15C	109.5
C3—C4—H4A	120.0	H15A—C15—H15C	109.5
C4—C5—C6	119.43 (8)	H15B—C15—H15C	109.5
C4—C5—H5A	120.3	N2—C16—C17	111.85 (9)
C6—C5—H5A	120.3	N2—C16—H16A	109.2
C5—C6—C1	121.00 (8)	C17—C16—H16A	109.2
C5—C6—N1	118.26 (8)	N2—C16—H16B	109.2
C1—C6—N1	120.73 (8)	C17—C16—H16B	109.2
N1—C7—C8	125.64 (8)	H16A—C16—H16B	107.9
N1—C7—H7A	117.2	C16—C17—H17A	109.5
C8—C7—H7A	117.2	C16—C17—H17B	109.5
C7—C8—C9	117.96 (8)	H17A—C17—H17B	109.5
C7—C8—C13	123.96 (8)	C16—C17—H17C	109.5
C9—C8—C13	118.04 (8)	H17A—C17—H17C	109.5
C10—C9—C8	121.68 (9)	H17B—C17—H17C	109.5
C10—C9—H9A	119.2		
			
C6—C1—C2—C3	−0.18 (14)	N1—C7—C8—C13	−6.89 (16)
C1—C2—C3—C4	0.32 (14)	C7—C8—C9—C10	177.47 (9)
C1—C2—C3—S1	177.54 (7)	C13—C8—C9—C10	−0.33 (15)
O1—S1—C3—C2	160.63 (8)	C8—C9—C10—C11	−1.77 (15)
O2—S1—C3—C2	38.60 (9)	C16—N2—C11—C10	177.74 (9)
O3—S1—C3—C2	−79.93 (8)	C14—N2—C11—C10	−4.37 (15)
O1—S1—C3—C4	−22.17 (9)	C16—N2—C11—C12	−0.73 (15)
O2—S1—C3—C4	−144.20 (8)	C14—N2—C11—C12	177.16 (9)
O3—S1—C3—C4	97.27 (8)	C9—C10—C11—N2	−175.28 (10)
C2—C3—C4—C5	0.28 (14)	C9—C10—C11—C12	3.24 (14)
S1—C3—C4—C5	−176.89 (7)	N2—C11—C12—C13	175.78 (9)
C3—C4—C5—C6	−1.00 (15)	C10—C11—C12—C13	−2.75 (14)
C4—C5—C6—C1	1.15 (15)	C11—C12—C13—C8	0.74 (15)
C4—C5—C6—N1	179.62 (9)	C7—C8—C13—C12	−176.80 (9)
C2—C1—C6—C5	−0.56 (14)	C9—C8—C13—C12	0.85 (14)
C2—C1—C6—N1	−178.99 (8)	C11—N2—C14—C15	84.88 (12)
C7—N1—C6—C5	153.95 (10)	C16—N2—C14—C15	−97.11 (11)
C7—N1—C6—C1	−27.57 (14)	C11—N2—C16—C17	86.61 (12)
C6—N1—C7—C8	172.99 (9)	C14—N2—C16—C17	−91.41 (11)
N1—C7—C8—C9	175.46 (10)		

### Hydrogen-bond geometry (Å, º)

**Table:**

*D*—H···*A*	*D*—H	H···*A*	*D*···*A*	*D*—H···*A*
N1—H1*N*1···O3^i^	0.865 (18)	2.466 (17)	3.0309 (12)	123.6 (13)
N1—H1*N*1···O3^ii^	0.865 (18)	2.076 (18)	2.8989 (12)	158.7 (16)
C7—H7*A*···O2^iii^	0.93	2.39	3.2719 (13)	157
C13—H13*A*···O3^ii^	0.93	2.40	3.3002 (13)	164
C14—H14*A*···O1^iv^	0.97	2.58	3.4805 (14)	155

Symmetry codes: (i) *x*+1, *y*, *z*; (ii) −*x*, −*y*+1, −*z*+1; (iii) −*x*, −*y*, −*z*+1; (iv) −*x*+1, −*y*, −*z*+1.
